# Effects of Silibinin on Delaying Aging in *Drosophila melanogaster*

**DOI:** 10.3390/antiox14020147

**Published:** 2025-01-27

**Authors:** Kai Zhu, Hang Ni, Eqra Hafeez, Yaxuan Hu, Fan Hu, Dongsheng Du, Dongsheng Chen

**Affiliations:** 1 Anhui Provincial Key Laboratory of Molecular Enzymology and Mechanism of Major Metabolic Diseases, College of Life Sciences, Anhui Normal University, Wuhu 241000, China; zhuk@ahnu.edu.cn (K.Z.); eqrahafeez@ahnu.edu.cn (E.H.); huyaxuan@ahnu.edu.cn (Y.H.); hf20010925@ahnu.edu.cn (F.H.); 2 Anhui Provincial Key Laboratory of Biodiversity Conservation and Ecological Security in the Yangtze River Basin, College of Life Sciences, Anhui Normal University, Wuhu 241000, China; nh730@ahnu.edu.cn

**Keywords:** silibinin, aging, *Drosophila melanogaster*, lifespan

## Abstract

Aging is an inevitable physiological process, but delaying aging has always been an enduring human pursuit. Silibinin (SIL), derived from the seeds of the milk thistle plant, exhibits a broad spectrum of pharmacological properties, including anti-tumor effects, liver protection, inhibition of apoptosis, and alleviation of inflammation. However, whether it has anti-aging effects remains unclear. The SIL dietary supplement to *Drosophila melanogaster* prolonged lifespan, improved climbing ability, ameliorated age-associated intestinal barrier disruption, enhanced the resistance to oxidative stress, and increased the enzyme activities of superoxide dismutase (SOD) and catalase (CAT). Furthermore, RNA-seq results showed that SIL addition significantly upregulated 74 genes and downregulated 50 genes compared with the control. KEGG (Kyoto Encyclopedia of genes and genomes) analysis demonstrated that these differentially expressed genes were primarily involved in the Toll signaling pathway and endoplasmic reticulum proteins processing, six among which, including *IM2*, *IM3*, *Drsl3*, *CG7556*, *GCS1,* and *TRAM*, were particularly involved in the regulation by SIL supplementation. The results indicate that SIL exhibits anti-aging effects by enhancing antioxidant capacity and regulating aging-related signaling pathways. Therefore, SIL shows a potential application in anti-aging dietary regimens.

## 1. Introduction

Aging, representing a natural physiological decline, is associated with the onset of cancer, diabetes, and cardiovascular and neurodegenerative diseases [1]. For a long time, the free radical theory (such as reactive oxygen species, ROS) has been thought to drive aging. The excessive accumulation of intracellular free radicals could lead to oxidative damage, which has a significant impact on lifespan [2]. Therefore, enhancements in the antioxidant system and reductions in ROS could prolong the lifespan [3].

In recent years, researchers have focused on the relationship between immunity and aging, giving rise to the immunological theory of aging. As a result of malfunctions of innate and adaptive immune responses, the body’s capacity to resist microorganisms and toxic substances that invade diminishes with age. During aging, the efficacy of adaptive immune response diminishes while innate immunity often becomes more hyperactive, leading to an increased susceptibility to inflammatory responses [4,5,6,7]. The above changes could inflict harm on the tissues, leading to accelerated aging and the onset of age-related diseases [8,9].

Given the intense interest in maintaining health and prolonging lifespan, increasing research, ranging from exercise regulation and dietary control to health product pharmacology, has been pursued to understand aging processes [10,11,12]. Many plant-derived extracts are important sources for the preparation of health drugs. It has been reported that plant extracts, such as *Angelica keiskei* extracts, gastrodin, *Agrocybe aegerita* polysaccharides, and ginsenosides, can prolong the lifespan of fruit flies [13,14,15,16]. Due to its short lifespan, strong reproductive ability, and clear genetic background, *Drosophila melanogaster* is an excellent model organism not only in studying developmental biology but also in studying aging [17,18,19,20].

**Figure 1:**
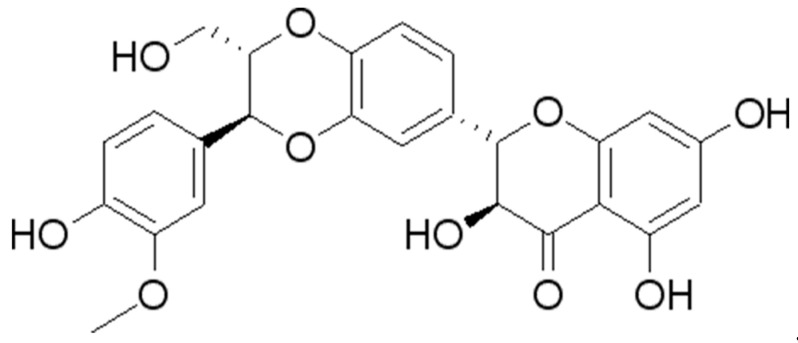
Chemical structure formula of SIL.

Silibinin (SIL) (Figure 1), a flavonoid substance, is the primary component of silymarin extract from the seeds of *Silybum marianum* (also called milk thistle). SIL exhibits a broad spectrum of pharmacological activities, such as anti-tumor, antioxidant, anti-inflammatory, liver-protecting, and cardiovascular-protecting functions [21,22,23,24,25,26,27]. Due to its excellent antioxidant activities, SIL functions as a scavenger of free radicals and has been widely used in the treatment of multiple diseases, such as hepatitis, sepsis, and cardiovascular disease [28,29,30,31]. To date, the anti-aging effects and the associated mechanisms of SIL in *Drosophila* remain unclear. In this study, we examined the effect of SIL in delaying aging in fruit flies.

## 2. Materials and Methods

### 2.1. Chemicals

SIL (Cat# S817884, purity of 98%) was purchased from Shanghai Macklin Biochemical Technology Co., Ltd. (Shanghai, China); the SOD assay kit (Cat# S0101S) and MDA assay kit (Cat# S0131S) were purchased from Beyotime Biotechnology Co., Ltd. (Shanghai, China); and the CAT assay kit (Cat# A007-1-1) was purchased from Nanjing Jiancheng Bioengineering Institute (Nanjing, China).

### 2.2. Drosophila Strain and Culture

All experiments in this study used male fruit flies unless specifically stated. All fly stocks were raised at 25 °C on a standard fly medium (Table 1), except those with special requirements. The *w*^1118^ strain was used as the wild-type strain and obtained from Bloomington *Drosophila* Stock Center (BDSC). SIL was first dissolved in a small amount of DMSO then added to the medium and thoroughly mixed. The concentrations of SIL were 0.6 mg/mL, 1.2 mg/mL, and 2.4 mg/mL. The flies were transferred to a fresh medium every 3 days.

### 2.3. Lifespan Assay

Three-day-old *w*^1118^ fruit flies were collected and reared on a standard medium (control group) and SIL-supplemented medium (treatment group), with 150 flies per group. To prevent overcrowding, each group of flies was cultured separately in six vials (25 flies per vial). The medium was replaced every 3 days, and the number of dead flies in each vial was recorded until all flies died. The lifespan curve was plotted for each group, and the mean, median, and maximum lifespan were calculated. There are three concentrations in the SIL treatment group, including 0.6 mg/mL, 1.2 mg/mL, and 2.4 mg/mL.

### 2.4. Food Intake Detection

Food intake detection was conducted based on Han’s method [32]. Sixty fruit flies in each group were reared on the standard medium (control group) and the medium supplemented with SIL (treatment group) for 7 days then transferred to the standard medium containing 2.5% (*w*/*v*) brilliant blue (dye, Macklin) for 12 h. Each group of flies was then divided into three subgroups (20 flies per subgroup), and each subgroup was homogenized and centrifuged in 1 mL of phosphate-buffered saline. The absorbance of the supernatant at 595 nm was measured using a spectrophotometer. Three values were obtained for each group, and the average was calculated, which reflects the feeding behavior of the fruit flies.

### 2.5. Body Weight Detection

Sixty fruit flies in each group were reared in either the standard medium (control group) or the medium supplemented with SIL (treatment group). On the 20th and 40th days, the fruit flies in each group were anesthetized with CO_2_, and then each group was divided into three subgroups (20 flies per subgroup). The 20 flies in each subgroup were weighed together, and then the total weight was divided by 20 to obtain the average weight of each fruit fly in that subgroup. Three values were obtained for each group, and then the average of these three values was calculated.

### 2.6. Climbing Assay

The locomotor ability was evaluated using a negative geotropism test [32]. Sixty fruit flies in each group were cultured in the medium without addition (control group) and the medium supplemented with SIL (treatment group) for 20 days and 40 days, respectively. After that, the fruit flies in each group were evenly divided into three subgroups (20 flies per subgroup), and the flies in each subgroup were placed in an empty culture tube and cultured at 25 °C for 30 min to acclimatize to the environment. Afterward, fruit flies were gently patted to the bottom of the tube, and the percentage of fruit flies that climbed to 5 cm or more within 15 s was recorded. This percentage was used as an indicator of the climbing ability of the fruit flies. Three values were obtained for each group, and their average was calculated.

### 2.7. Smurf Assay

The “Smurf” assay commonly uses a non-absorbable dye (bright blue, Macklin) to evaluate the barrier function of the fruit fly gut [33,34]. Male flies were reared for 20 and 40 days in medium without (control group) and with SIL (treatment group) (300 flies per group). Subsequently, each group of flies was divided into three subgroups (100 flies per subgroup), starved for 1 h, and then transferred to food containing 2.5% *w*/*v* blue dye for 2.5 h. The percentage of “Smurf” flies was then calculated, with flies whose entire bodies were stained blue being classified as “Smurf” flies. Three values were obtained for each group (three subgroups), and the average was calculated.

### 2.8. Hydrogen Peroxide (H_2_O_2_) Challenge Assay

H_2_O_2_ was used to generate hydroxyl radicals (·OH). After H_2_O_2_ induction, the level of ROS in flies increased, resulting in accelerated aging. The method was adapted from Liu et al. [35]. Newly hatched male flies were collected and reared in a medium without (control group) and with SIL (treatment group). A total of 100 fruit flies were used in each group and evenly distributed in 5 tubes for cultivation (20 flies per tube). On the 20th day, the fruit flies were placed in empty rearing tubes with filter paper soaked in distilled water and starved for 2 h and then transferred to test tubes with filter paper soaked in a 6% sucrose–30% H_2_O_2_ solution. The death of fruit flies was recorded every 2 h until all of the flies died. Using the death data of all of the flies in each group, the lifespan curve was plotted.

### 2.9. Intensive Paraquat Challenge Assay

Paraquat can generate superoxide anions, causing oxidative damage to fruit flies. Newly emerged male fruit flies were reared in a standard medium (control group) and a medium supplemented with SIL (treatment group) for 20 days. A total of 100 fruit flies were used in each group and evenly distributed in 5 tubes for cultivation (20 flies per tube). After fasting for 2 h, they were transferred to filter paper soaked in a 20 mM paraquat solution (containing 6% sucrose). The death of flies was recorded every 2 h until all of the fruit flies died. Using the death data of all the fruit flies in each group, a lifespan curve was plotted.

### 2.10. Antioxidant Enzyme Activities and MDA Content

Newly hatched male flies were reared in a standard medium and media supplemented with 0.6 mg/mL, 1.2 mg/mL, and 2.4 mg/mL of SIL (treatment group) (90 fruit flies in each group). On the 40th day, the fruit flies in each group were evenly divided into three subgroups (30 flies per subgroup). After being starved for 1 h, the 30 fruit flies in each subgroup were quickly frozen in liquid nitrogen. Subsequently, the frozen samples were homogenized in phosphate-buffered solution and centrifuged at 1500× *g* for 10 min at 4 °C. The supernatant was collected and stored at −80 °C. The activities of superoxide dismutase (SOD) and catalase (CAT) and the content of malondialdehyde (MDA) in each group of samples were measured. Three values were obtained for each group, and then the average was calculated.

### 2.11. RNA-Seq

RNA-seq was conducted by Novogene Bioinformatics Technology Co., Ltd. (Beijing, China). Male flies were cultured in standard foods (control) or a medium supplemented with 1.2 mg/mL SIL for 30 days. Total RNA was independently extracted from *Drosophila* whole body using the Trizol Reagent (Sangon Biotech, Shanghai, China). The purity and concentration of RNA were measured using the Agilent Bioanalyzer 2100 system. The sequencing library was constructed and sequenced on an Illumina platform. The raw reads were filtered. Simultaneously, the Q20 and Q30 of clean reads were calculated. DESeq2 was used to perform differential expression analysis. The statistical enrichment of the Gene Ontology (GO) and KEGG pathways of differentially expressed genes were analyzed using the clusterProfile software (Version 3.5.0).

### 2.12. Real-Time PCR

Flies were reared in standard (control) or SIL-supplemented food for 40 days and then frozen in liquid nitrogen. Total RNA was extracted, and the cDNA was prepared using PrimeScript™ RT reagent kit with gDNA Eraser (TAKARA). CT values were calculated to analyze the relative expression of genes. The following primers were used to amplify differential expression genes (*rp49* gene as reference) (Table 2).

### 2.13. Statistical Analyses

Data are expressed as mean ± standard deviation (S.D). Statistical analysis was performed using GraphPad Prism6 (Version No. 6, GraphPad Software, La Jolla, CA, USA). In addition to survival, statistical significance was established using one-way analysis of variance (ANOVA) and t-test. The survival rates among the groups were compared, and the logrank test was used for the significance test. For all analyses, significant levels of *p* values were expressed as * < 0.05, ** < 0.01, *** < 0.001, and **** < 0.0001.

## 3. Results

### 3.1. SIL Supplementation Extends the Lifespan in Drosophila

**Figure 2:**
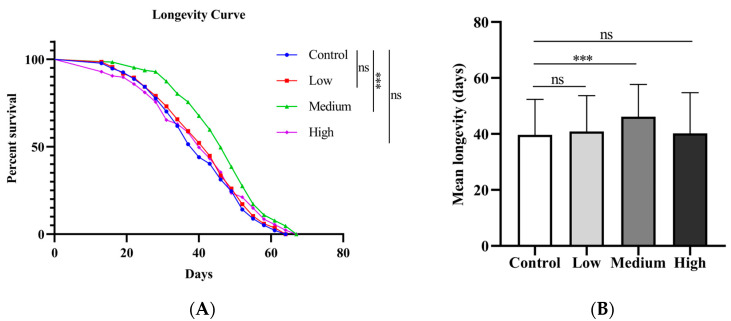
Effects of SIL on longevity. (**A**) Survival curves and (**B**) average lifespans of *Drosophila melanogaster* treated with different concentrations of SIL (150 flies were used in each group). Low, medium, and high refer to 0.6 mg/mL, 1.2 mg/mL, and 2.4 mg/mL of SIL, respectively. ns represents not significant; *** *p* < 0.001.

To explore whether SIL prolongs the lifespan of fruit flies, we performed an assay using male *w*^1118^ as experimental material by adding SIL to the basic culture medium. In this experiment, three concentrations of SIL (0.6 mg/mL, 1.2 mg/mL, and 2.4 mg/mL) were used to examine the lifespan of fruit flies. We found that, compared with the control group (no dietary addition of SIL), the mean lifespan of flies fed with 1.2 mg/mL SIL was extended by 16% (*p* < 0.001) (Figure 2A,B; Table 3). However, both lower and higher concentrations of SIL did not affect the lifespan of male flies. The results suggest that SIL supplementation extends the lifespan of *Drosophila* in a concentration-dependent manner. We speculate that high concentrations of SIL may cause toxicity to fruit flies and shorten their lifespan instead.

Furthermore, to examine whether there was a gender-related difference in the lifespan-extending effects of SIL in *Drosophila*, we investigated the impact of different concentrations of SIL on the lifespan of female fruit flies. The results showed that 1.2 mg/mL SIL significantly prolonged the lifespan of female *Drosophila*, which was consistent with the effect on males (Supplementary Figure S1). Taken together, the above results indicate that the lifespan-extending effects of SIL do not differ between sexes. Therefore, male *Drosophila* were selected for subsequent experiments.

### 3.2. Sil Fails to Affect Food Intake and Body Weight of Flies

Caloric restriction (CR) has been one of the most effective strategies for extending the lifespan of animals [36,37]. We are uncertain whether the SIL addition will decrease the food consumption of fruit flies, thus prolonging their lifespan. Therefore, it is necessary to measure the food intake of flies after SIL supplementation.

**Figure 3:**
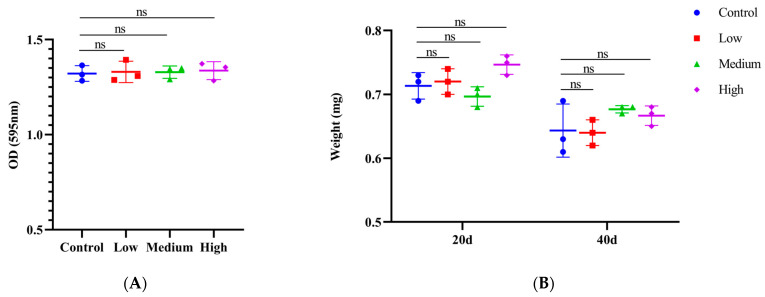
Effects of SIL on feeding behavior and body weight in *Drosophila.* (**A**) Effects of different concentrations of SIL on food intake. (**B**) The detection of fly body weight after treated with three concentrations of SIL at days 20 and 40. In the dot plot, each dot represents the value of an independent experiment (including 20 fruit flies). Low, medium, and high refer to 0.6 mg/mL, 1.2 mg/mL, and 2.4 mg/mL of SIL, respectively. ns represents not significant.

In food intake assays, it is common to reflect changes in dietary behavior by adding bright blue to the food in *Drosophila*. The results showed that there was no difference between the SIL-feeding group with different concentrations (0.6 mg/mL, 1.2 mg/mL, and 2.4 mg/mL) and the control group (Figure 3A). This result indicates that SIL does not influence fruit flies’ food intake, and the lifespan extension of SIL is not achieved through CR in *Drosophila*.

In animals, the body weight is frequently influenced by their dietary behavior. Therefore, we assessed the fly weight after administering SIL. The results showed that, compared with the control, there was no difference in body weight after the continuous supplementation of SIL for 20 and 40 days (Figure 3B). Taken together, we eliminated the effects of CR on lifespan in *Drosophila*.

### 3.3. SIL Improves the Locomotor Ability

**Figure 4:**
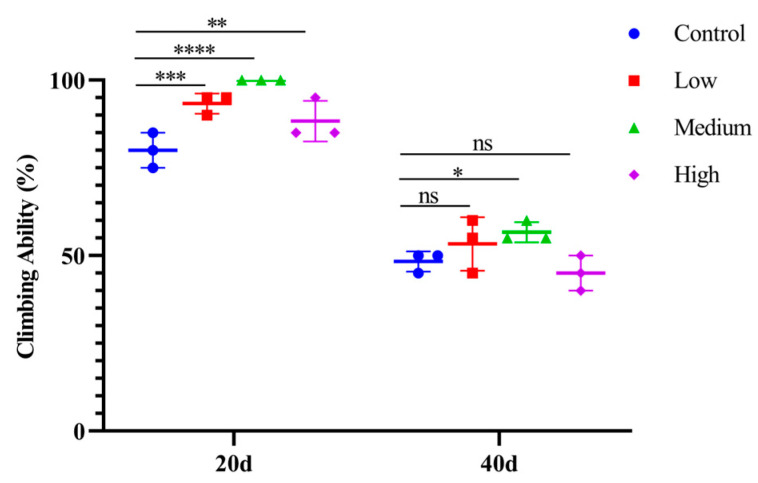
The climbing ability of fruit flies treated with different concentrations of SIL at days 20 and 40. In the dot plot, each dot represents the value of an independent experiment (including 20 fruit flies). Low, medium, and high refer to 0.6 mg/mL, 1.2 mg/mL, and 2.4 mg/mL of SIL, respectively. ns represents not significant; * *p* < 0.05; ** *p* < 0.01; *** *p* < 0.001; **** *p* < 0.0001.

Fruit flies exhibit a habit of climbing upwards in enclosed spaces [38], so the climbing ability is an important indicator of insect motility. The climbing ability can be quantified by recording the number of fruit flies that climb to a set point within a specified time. As shown in Figure 4, compared with 20-day-old flies, the climbing ability of each group collected from 40-day-old flies (with or without SIL added) was significantly decreased, suggesting that athletic ability is closely related to aging.

We subsequently analyzed the locomotor ability of fruit flies that were continuously supplemented with SIL for 20 and 40 days, using the group without SIL addition as the control. The results showed that, compared to the control, the climbing ability of flies fed with SIL for 20 days was significantly increased; the middle-concentration group (1.2 mg/mL) increased the most by 25.0% (*p* < 0.0001) (Figure 4). With continuous SIL supplementation for 40 days, the improvement of climbing ability was only observed in the middle-concentration group, with an increase of 17.26% (*p* < 0.05) (Figure 4). The results suggest that SIL enhances athletic ability in both an age-dependent and a concentration-dependent manner in *Drosophila*.

### 3.4. SIL Prevents the Intestinal Barrier Dysfunction in Aged Flies

**Figure 5:**
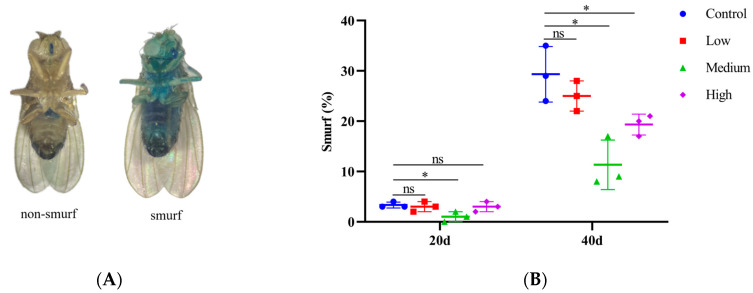
Effects of SIL on intestinal integrity. (**A**) Non-Smurf and Smurf flies. (**B**) The proportions of Smurf flies treated with different concentrations of SIL at days 20 and 40. In the dot plot, each dot represents the value of an independent experiment (including 100 fruit flies). Low, medium, and high refer to 0.6 mg/mL, 1.2 mg/mL, and 2.4 mg/mL of SIL, respectively. ns represents not significant; * *p* < 0.05.

Previous studies have shown that the gut of young fruit flies maintains structural integrity and low intestinal permeability by relying on tissue homeostasis and cell junctions. Conversely, the intestinal barrier protection in older fruit flies is compromised, resulting in increased permeability [39,40]. As shown in Figure 5A, in fruit flies with intact intestinal function after consuming blue dye food, the blue coloration was largely confined to the mouthpart and the digestive tract. However, when the intestinal barrier function was impaired, the blue dye became clearly visible throughout the entire body, leading to the nickname “Smurfs” [40].

To explore the physiological protective effects of SIL on the gut of older flies, we performed the “Smurfs” assay to assess the integrity of the physical barrier in the gut of fruit flies. As shown in Figure 5B, in 20-day-old *Drosophila*, the percentage of Smurf flies in the group treated with 1.2 mg/mL SIL significantly decreased. With increasing age, the number of Smurf flies in each group (including the control group and the SIL treatment groups) significantly increased. In 40-day-old *Drosophila*, the percentages of Smurf flies in the 1.2 mg/mL and 2.4 mg/mL SIL treatment groups significantly decreased. Moreover, the 1.2 mg/mL SIL treatment group exhibited the most significant reduction, with a decrease of 61.37%. These findings fully demonstrate that SIL supplementation prevents intestinal barrier dysfunction in old flies, thereby preserving the integrity of intestinal tracts.

### 3.5. SIL Enhances the Antioxidant Capacity

**Figure 6:**
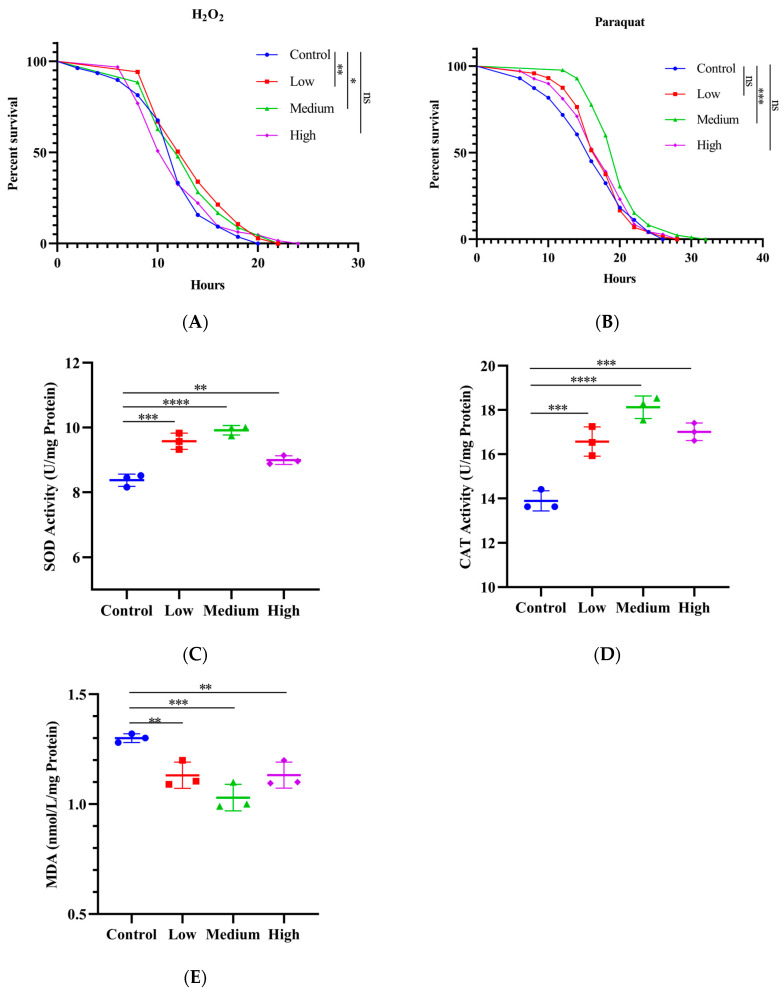
Effects of SIL on antioxidant ability in fruit flies. Survival curves of flies treated with SIL under H_2_O_2_-induced (**A**) and paraquat-induced (**B**) oxidative stress (100 flies were used in each group). (**C**) The SOD activity, (**D**) CAT activity, and (**E**) MDA content of flies treated with different concentrations of SIL for 40 days. In the dot plot, each dot represents the value of an independent experiment (including 30 fruit flies). Low, medium, and high refer to 0.6 mg/mL, 1.2 mg/mL, and 2.4 mg/mL of SIL, respectively. ns represents not significant; * *p* < 0.05; ** *p* < 0.01; *** *p* < 0.001; **** *p* < 0.0001.

Numerous plant extracts, such as polysaccharides, oligosaccharides, anthocyanins, and saponins, can extend the lifespan of flies by enhancing their resistance to oxidative stress [41,42,43,44]. To determine whether SIL extends the lifespan of flies by enhancing the antioxidant capacity, male fruit flies were fed with SIL for 20 days and then transferred to filter paper containing 30% H_2_O_2_ and 20 mM paraquat solution to record the mortality. The results showed that SIL supplementation notably boosted survival rates under H_2_O_2_- and paraquat-induced oxidative stress conditions compared with the control group (Figure 6A,B). The average survival rates of the 0.6 mg/mL and 1.2 mg/mL SIL treatment groups in H_2_O_2_ stress rose by 15.24% and 11.35%, respectively, compared to the control group. Meanwhile, the average survival rates of the 1.2 mg/mL SIL treatment groups in paraquat stress rose by 23.40% compared with the control.

To explore whether SIL extends the lifespan by enhancing the activity of antioxidant enzymes in flies, we detected the activities of the SOD and CAT in male flies treated by SIL. The SOD activity was significantly increased in flies treated with three concentrations (0.6 mg/mL, 1.2 mg/mL, and 2.4 mg/mL) of SIL for 40 days (Figure 6C). The activity of CAT was also markedly increased by the same dose of SIL supplementation (Figure 6D). Malondialdehyde (MDA) is the product of free radical reactions, which directly reflects the degree of oxidative damage in organisms [45]. As shown in Figure 6E, the levels of MDA were significantly reduced under different concentrations of SIL treatment. The above results indicate that SIL exerts anti-aging effects by enhancing the antioxidant capacity in fruit flies.

### 3.6. SIL Inhibits Toll Signaling Pathway and Activates ER Proteins Processing-Related Pathway in Flies

**Figure 7:**
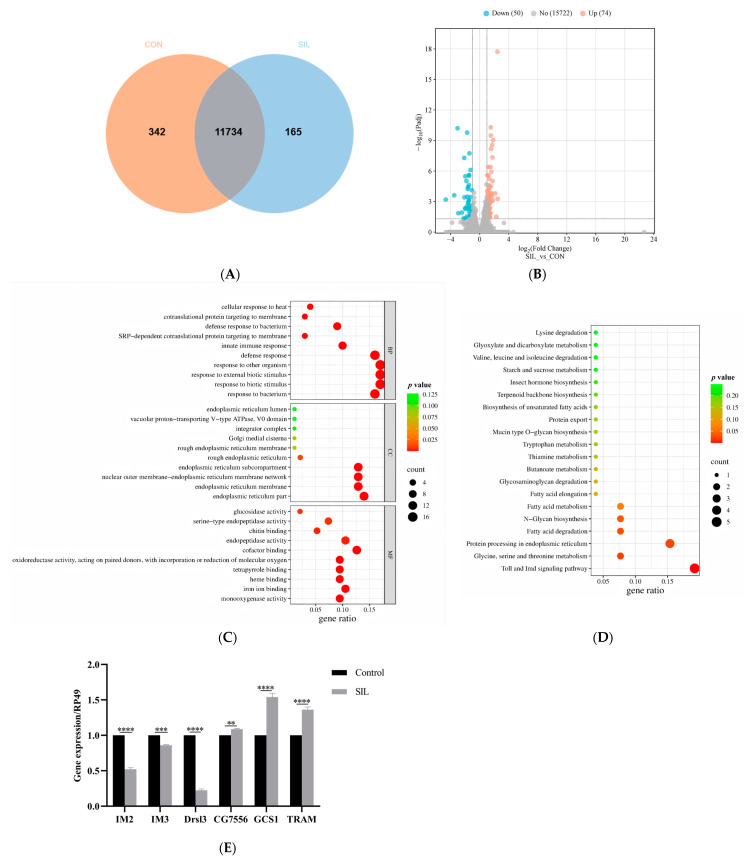
Effects of SIL on gene expression in 30-day-old fruit flies. (**A**) Venn diagram and (**B**) volcano plots containing the number of differentially expressed genes between the flies treated with or without SIL. (**C**) GO and (**D**) KEGG pathway analysis of differentially expressed genes between the flies treated with or without SIL. (**E**) Validation of six differentially expressed genes using qRT-PCR (the assay was repeated 3 times across different replicates, 10 flies were used for each time). ** *p* < 0.01; *** *p* < 0.001; **** *p* < 0.0001.

To identify key functional gene sets that may be associated with SIL anti-aging, we performed RNA sequencing (RNA-Seq) on 30-day-old fruit flies treated without (as control) and with SIL (1.2 mg/mL). The expression profiles of approximately 12,000 genes were obtained from the sequencing database, and further bioinformatic analysis showed that more than 80% of these genes were expressed in both groups (Figure 7A). The transcript levels of 124 key genes were significantly altered by long-term treatment with SIL. Among them, 74 genes were upregulated and 50 genes were downregulated in SIL-treated groups compared with control groups (Figure 7B and Supplementary Datasets S1 and S2). To explore the biological functions of differentially expressed genes, we conducted Gene Ontology (GO) functional enrichment analysis. The results showed that altered genes by SIL were mainly involved in biological processes, such as responses to the bacterium, responses to biotic stimulus, defense responses, innate immune responses, cotranslational protein targeting membranes, and cellular responses to heat (Figure 7C and Supplementary Dataset S3).

Kyoto Encyclopedia of Genes and Genomes (KEGG) pathway enrichment analyses revealed that differentially expressed mRNAs were mainly involved in five pathways, including the Toll signaling pathway; glycine, serine, and threonine metabolism; protein processing in the endoplasmic reticulum (ER); fatty acid degradation; and N-glycan biosynthesis (Figure 7D and Supplementary Dataset S4). The Toll signaling pathway was downregulated significantly, suggesting that supplementation with SIL inhibited the activity of this signaling pathway, while the protein processing in ER was markedly upregulated following SIL intervention (Supplementary Dataset S4), suggesting that the SIL diet promoted the physiological function of ER.

To further validate the expression profile of genes related to SIL’s anti-aging effects, we measured the expression of six genes associated with the Toll signaling pathway and ER protein processing using the qRT-PCR technique. The results showed that the mRNA expression levels of *IM2*, *IM3,* and *Drsl3* from the Toll signaling pathway were significantly reduced compared to the control group, while *CG7556*, *GCS1,* and *TRAM* (ER protein processing) expressions were significantly higher than those in the control group (Figure 7E). Taken together, these results indicate that SIL supplementation inhibits the activation of Toll while promoting protein processing in ER in aged flies.

## 4. Discussion

Aging is a progressive decline in physiological functions that accompanies the senescence of organisms, and the incidence of many diseases increases with age [46]. What is the mechanism of aging? How can we delay aging? These questions are hot topics for scientists to investigate. Natural compounds derived from plants have been recognized as potential agents for delaying aging and treating aging-related diseases [47]. *Drosophila melanogaster* is a model organism widely used in studying disease- and aging-related mechanisms, as well as in drug screening. Here, we explored the anti-aging effects of SIL in *Drosophila melanogaster*. The dietary addition of SIL significantly improved the lifespan, motility, and intestinal integrity in fruit flies, while there was no difference in food intake and body weight, indicating that the anti-aging effect of SIL is not due to dietary restrictions.

In diverse biological models, lifespan extension is closely associated with enhanced stress tolerance. Numerous environmental stress factors can stimulate organisms to generate excessive ROS. This, in turn, induces cellular oxidative stress, disrupts the normal redox balance, and leads to various adverse effects, ultimately accelerating aging [48,49]. El Assar et al. have demonstrated that, due to the decreased antioxidant capacity of the body, free radicals cannot be cleared in a timely manner, ultimately leading to inflammation and even death [50]. A decline in the activity of antioxidant enzymes, such as SOD and CAT, accelerates aging in fruit flies, whereas an increase in their activity can extend their lifespan [51,52]. Kumar et al. revealed that SIL can reduce oxidative damage and prolong nematode lifespan [53]. Our findings indicate that SIL can enhance the activity of SOD and CAT. Consequently, it significantly extends the lifespan of fruit flies under H_2_O_2_ and paraquat stress, which is consistent with the reports on nematodes.

Surprisingly, during RNA-Seq analysis, we found that the expression of SOD/CAT protein-coding genes did not increase significantly. This finding suggests that SIL enhances the activity of SOD/CAT enzymes in aged *Drosophila* through a non-transcriptional regulatory mechanism. There are at least two possibilities. First, SIL may regulate the translation of SOD/CAT proteins to increase the expression level of SOD/CAT enzymes. Second, SIL may directly bind to SOD/CAT proteins to enhance their activity.

Numerous studies have demonstrated that dietary supplements, such as purple sweet potato anthocyanins and apple polyphenols, can enhance the activity of antioxidant enzymes and prolong the lifespan of animals [32,54]. SIL is a flavonoid substance, indicating that flavonoids may have anti-aging effects by scavenging free radicals. In addition to clearing intracellular free radicals, SIL also possesses numerous other biological functions. For instance, it can inhibit the activation of poly (ADP-ribose)-polymerase (PARP), ultimately leading to the restoration of NAD^+^ levels, SIRT1 activity, and AMPK phosphorylation levels [29]. Liu et al. found that SIL can reduce the apoptosis of hippocampal neurons in over-trained rats, delay cell aging, and alleviate learning and memory impairment in rats [55]. Guo et al. demonstrated that SIL can improve H_2_O_2_-induced apoptosis of trophoblast cells and enhance the oxidative stress response by activating the Nrf2 signaling pathway [28]. The current research shows that SIL can improve the intestinal inflammatory response of fruit flies by regulating the c-Jun N-terminal kinase (JNK) signaling pathway [30].

ER plays a crucial role in protein folding, modification, and quality control. Therefore, some interventions that promote protein processing and ER homeostasis maintenance can extend lifespan [56]. When the protein-processing pathway in ER is disrupted, unfolded or misfolded proteins accumulate, which triggers ER stress, promotes apoptosis, and accelerates aging. Omics analysis has found that SIL can promote the processing of ER proteins; therefore, it can delay the aging of *Drosophila*. The Toll signaling pathway, first discovered in *Drosophila*, is crucial for embryonic development and immune defense. In recent years, it has been revealed that it is closely associated with aging. Aging causes the hyperactivation of the Toll signaling pathway, which prompts adaptor proteins like MyD88 to regulate the entry of NF-κB into the nucleus. Eventually, this leads to the massive expression of regulatory inflammatory factors, thus accelerating the aging process [57]. Moreover, the activation of the Toll pathway increases the production of ROS, which can damage cells and accelerate the aging process [58]. Here, we found that SIL addition can inhibit the activity of the Toll signaling pathway in aged fruit flies and promote the expression of processing proteins in ER. These results indicate that SIL is involved in numerous biological processes. However, whether these signaling pathways and biological processes regulated by SIL are more or less involved in regulating the aging of animals? These are topics worthy of further study in the future.

## 5. Conclusions

The objective of this study was to investigate the effects of dietary supplementation with SIL on anti-aging in *Drosophila melanogaster*. The SIL supplementation dramatically extended lifespan, improved the locomotor ability, ameliorated age-related intestinal barrier damage, and enhanced the antioxidant ability. The food intake and body weight were not affected in flies treated with SIL. The Toll signaling pathway was found to be inhibited, while the ER protein-processing-related pathway was activated.

The results indicate that SIL strongly exhibits anti-aging effects by enhancing antioxidant capacity and regulating aging-related signaling pathways, and, therefore, SIL shows potential application in the production of functional food products.

## Figures and Tables

**Table 1:** The ingredients of the standard *Drosophila* medium.

Ingredients	Proportions
Corn powder	75.0 g/L
Sucrose	30.0 g/L
Yeast	25.0 g/L
Agar	10.0 g/L
Methylparaben	1.5 g/L
Propionic acid	2 mL/L

**Table 2:** The primers used for real-time PCR.

Gene Name	Forward Primer	Reverse Primer	Gene Accession Number
IM2	AGTCGTCACCGTCTTTGTGTT	CAGTATTTGCAGTCCCCGTTG	NM_166277.3
IM3	TCACTCGCCTTCGTTTTGGG	TTAGGCCCTCACATTGCAGAC	NM_001299640.1
Drsl3	GTTTTGGCACGTGATTGCCT	TCCACTGACATGTCCCTCCT	NM_168020.3
CG7556	GAGACCAACTGGACGCAAGA	GGCACTCCTCCTTGGTCTTC	NM_001298498.1
GCS1	ACTTCGGCATGAAGACCAGG	TCTTGAACGCCAAAACTGCG	NM_132479.3
TRAM	TGGCCATTAAACCGGGACTC	GACGTTGTGGTGCAGGGATA	NM_166867.2
rp49	CCAGTCGGATCGATATGCTA	GTTGTGCACCAGGAACTTCT	NM_170460.2

**Table 3:** The effects of SIL on the lifespan of *Drosophila melanogaster*.

	Mean (±SEM, d)	Maximum (d)	Median (d)
Control	39.71 ± 1.09	64	40
Low	40.92 ± 1.11	64	43
Medium	46.19 ± 1.02 ^a^	67	46
High	40.21 ± 1.29	67	40

^a^, *p* < 0.001 vs. control.

## Data Availability

The data that support the findings of this study are available from the corresponding author upon reasonable request.

## Supplementary Materials

The following supporting information can be downloaded at: https://www.mdpi.com/article/10.3390/antiox14020147/s1, Supplementary Figure S1: Survival curves of female *Drosophila* fed with different concentrations of SIL; Supplementary Dataset S1: The 74 genes upregulated in the SIL-treated groups compared with control groups; Supplementary Dataset S2: The 50 genes downregulated in the SIL-treated groups compared with control groups; Supplementary Dataset S3: The GO analysis of differentially expressed genes between the flies treated with or without SIL; Supplementary Dataset S4: The KEGG pathway analysis of differentially expressed genes between the flies treated with or without SIL.
