# Supplementary material for: Effects of Silibinin on Delaying Aging in Drosophila melanogaster
Source: Antioxidants (Basel). 2025 Jan 27;14(2):147. doi: 10.3390/antiox14020147 (PMC11851952; doi:10.3390/antiox14020147)
Supplement: Supplementary file 1 [file antioxidants-14-00147-s001.zip › Supplementary Figure S1.pdf]

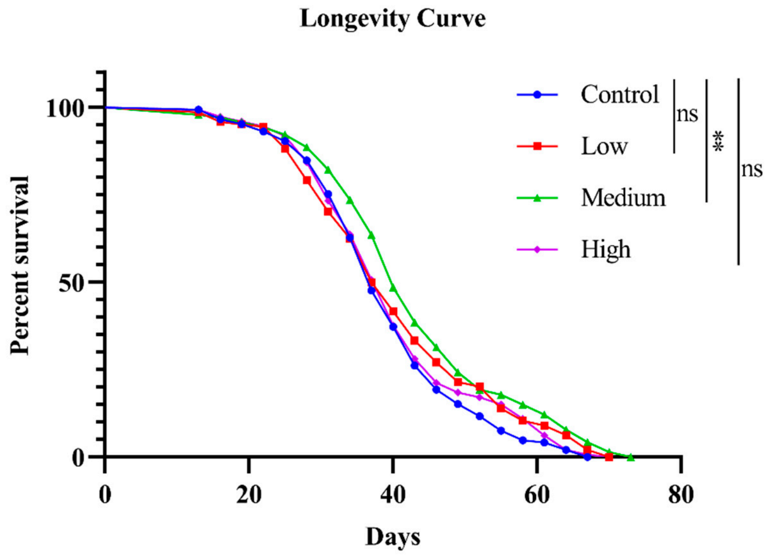

Supplementary Figure S1. Survival curves of female *Drosophila* fed with different concentrations of SIL (150 flies were used in each group). $^{**}p < 0.01$.
